# Organophosphorus Nerve Agents: Types, Toxicity, and Treatments

**DOI:** 10.1155/2020/3007984

**Published:** 2020-09-22

**Authors:** Sudisha Mukherjee, Rinkoo Devi Gupta

**Affiliations:** Faculty of Life Sciences and Biotechnology, South Asian University, New Delhi 110021, India

## Abstract

Organophosphorus compounds are extensively used worldwide as pesticides which cause great hazards to human health. Nerve agents, a subcategory of the organophosphorus compounds, have been produced and used during wars, and they have also been used in terrorist activities. These compounds possess physiological threats by interacting and inhibiting acetylcholinesterase enzyme which leads to the cholinergic crisis. After a general introduction, this review elucidates the mechanisms underlying cholinergic and noncholinergic effects of organophosphorus compounds. The conceivable treatment strategies for organophosphate poisoning are different types of bioscavengers which include stoichiometric, catalytic, and pseudocatalytic. The current research on the promising treatments specifically the catalytic bioscavengers including several wild-type organophosphate hydrolases such as paraoxonase and phosphotriesterase, phosphotriesterase-like lactonase, methyl parathion hydrolase, organophosphate acid anhydrolase, diisopropyl fluorophosphatase, human triphosphate nucleotidohydrolase, and senescence marker protein has been widely discussed. Organophosphorus compounds are reported to be the nonphysiological substrate for many mammalian organophosphate hydrolysing enzymes; therefore, the efficiency of these enzymes toward these compounds is inadequate. Hence, studies have been conducted to create mutants with an enhanced rate of hydrolysis and high specificity. Several mutants have been created by applying directed molecular evolution and/or targeted mutagenesis, and catalytic efficiency has been characterized. Generally, organophosphorus compounds are chiral in nature. The development of mutant enzymes for providing superior stereoselective degradation of toxic organophosphorus compounds has also been widely accounted for in this review. Existing enzymes have shown limited efficiency; hence, more effective treatment strategies have also been critically analyzed.

## 1. Introduction

The usage of pesticides for suicidal poisoning resulted in ∼110,000 deaths each year during the span of 2010 to 2014, comprising 13.7% of total global suicides [1]. In India, the toxicity of these pesticides came into prominence around the year 1962 [2], which is estimated to be ∼168,000 deaths annually, that is, 19.7% of global suicides [1]. Extensive exposure to organophosphorus pesticides leads to various complications such as acute myocardial injury [3], cognitive impairment [4], and neuropsychiatric disorders in farmers [5]. Some organophosphorus pesticides also bioaccumulate in the environment [6], cause harm to birds such as vultures [7], and severely affect aquatic lifeforms [8] such as *Senegalese sole* and *Solea senegalensis* [9]. In Iran, Japan, and other terrorist attacks [10] including the assassination of Kim Jong-nam in Malaysia [11], these compounds were used as warfare agents, due to which, many international protocol and treaties have been entrenched. The Organisation for the Prohibition of Chemical Weapons (OPCW) was formed for implementing the Chemical Weapons Convention, which came in force on 29 April 1997. This body with its 193 member states supervises the global venture to permanently or verifiably eliminate chemical weapons [12].

## 2. Types of Organophosphorus Compounds

Organophosphorus compounds (OPCs) are organic chemicals derived from phosphoric acids and its derivatives and contain at least one carbon-phosphorus bond. The pentavalent types of phosphorus-containing compounds are primarily used in industrial and environmental applications. The substituents attached to the phosphorus of these esters of phosphoric acids play a vital role in toxicity [13]. Organophosphorus pesticides are thiols, amides, or esters of phosphonic, phosphinic, phosphoric, or thiophosphoric acids with two additional organic side chains of the phenoxy, cyanide, or thiocyanate group [14]. Some of the OPCs belong to the phosphonothioates (S-substituted), and phosphonofluoridate categories comprise of nerve agents, commonly known as chemical warfare agents [15].

**Figure 1:**
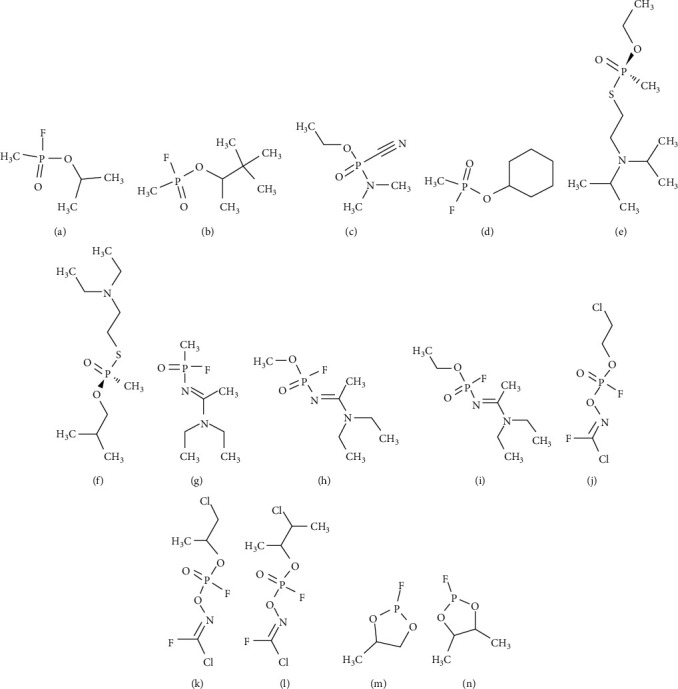
Chemical structure of G-series agents: (a) sarin, (b) soman, (c) tabun, and (d) cyclosarin; (e) V-series agent, VX; (f) VR (substance-33); chemical structures of A-series according to Dr. Mirzayanov: (g) A230, (h) A232, and (i) A234; chemical structures of A-series according Hoenig: (j) A230, (k) A232, and (l) A234; plausible and speculated chemical structures of Novichok: (m) Novichok-5 and (n) Novichok-7. For creating the chemical structures, ChemSketch software was used.

These nerve agents can be classified into four types: (1) the G-series agents which were developed by Germans and include tabun (GA), sarin (GB), soman (GD), and cyclosarin (GF) (Figures 1(a)–1(d)). (2) V-series agents, where V stands for venomous, include VE, VG, VM, and VX [11], and Chinese VX and Russian VX [16] (Figure 1(e)). (3) GV-series which have the combined properties of both series G and V, for example, GV, 2-dimethylaminoethyl-(dimethylamido)-fluorophosphate [17]. Generally, the G-series agents are less toxic compared to the V series [18]; (4) Novichok series of compounds, for example, substance-33, A230, A232, A234, Novichok-5, and Novichok-7 [19, 20]. Dr. Mirzayanov was first person to detail the development of the first three compounds, namely, substance-33, A230, and A232 at the GosNIIOKhT facility, Russia [21]. These compounds were unitary agents. Later, in the year 1989, the first Binary agent known as Novichok-5 was synthesised with Unitary A232 as its base structure [21]. Novichok agents are liquid in form, although they can convert into dusty formulation by the adsorption of liquid droplets on carriers such as silica gel, pumice, fuller's earth, or talc [22]. Hydrolysis of A230, A232, and A234 was seen to be slower in comparison to G- and V-series agents [23]. Generally owing to the secrecy surrounding their research, there is a huge debate regarding the structures of these compounds, and so, many different versions of structures have been speculated (Figures 1(f)–1(n)).

The fluoride-releasing volatile soman and sarin, cyanide-releasing tabun, and the thiocholine-releasing VX have a stereogenic phosphorus atom. All of these OPCs have two enantiomers P(−) and P(+), except for Soman which has two chiral atoms, one being a carbon centre and other being the phosphorus, which has four enantiomeric forms: C (+)P(+), C (+)P(−), C (−)P(+), and C (−)P(−) [24]. Comprehensive structural information regarding different types and isomers of the OPC nerve agents has been recently compiled and thoroughly reviewed [13, 15, 25, 26]. Stereoisomers play a crucial role in the context of the range of toxicity of the compound. Generally, P(−) enantiomers are more toxic in nature [27].

## 3. Physiological Effects

OPCs cross the respiratory epithelial and dermal membrane easily and gets distributed, especially in fat tissues. In suicidal cases, the absorptions through gastric mucosa have been reported. Most of these OPCs are inactive in their native form which converts to their active forms by a biotransformation process. This biotransformation process happens through oxidation of different groups such as sulphur group (e.g., parathion and malathion), thioether (e.g., disulfoton), amides (e.g., schradan and dichrotophos), and hydroxylation of the alkyl group (e.g., triorthocresylphosphate). Some OPCs may also undergo nonoxidative alteration to form toxic compounds as in the case of insecticides Trichlorfon and Nale [28]. After, the biotransformation, these OPCs can interact and inhibit the AChE which leads to the cholinergic crisis. In addition, it also interacts with certain biomolecules other than the AChE. Hence, the toxicity of OPCs has been divided into two categories, i.e., cholinergic and noncholinergic effects which have been discussed below in detail.

### 3.1. Cholinergic Effect

**Figure 2:**
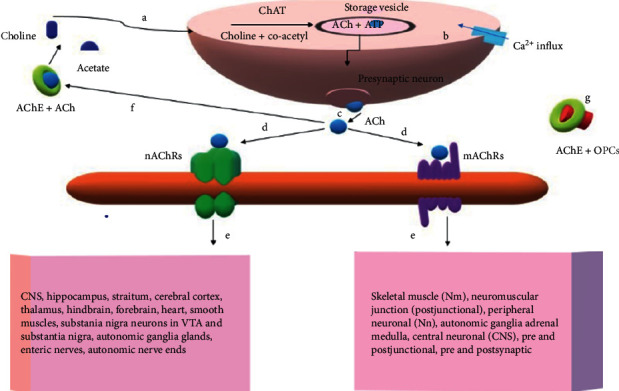
Depiction of cellular interaction of AChE, Ach, and OPCs. (a) Choline released from ACh hydrolysis, moves to axon where it reacts with acetyl moiety of co-acetyl to from ACh by an enzyme ChAT (choline acetyltransferase) and gets stored in vesicles along with cotransmitters such as ATP. (b) The influx of calcium results in the fusion of membranes. (c) Fusion leads to release of ACh into neuron junctions. (d) Interaction of ACh with nAChRs and mAChRs. (e) Signalling to different physiological targets. (f) Excess ACh after signalling, interacts with AChE and degrades into choline and acetate. (g) OPC binds with AChE and leads to increased ACh and endless signalling.

Acetylcholine (ACh) is a neurotransmitter [29] and interacts with the muscarinic acetylcholine receptors (mAChRs) which use G-protein signalling pathways [30]. This interaction has shown to play a vital role in the regulation of heart rate, contraction of smooth muscles, secretion of glands, and other CNS fundamental functions [30, 31]. ACh also interacts with nicotinic acetylcholine receptors (nAChRs) [30] (Figure 2) which have 5 subunits [32]. nAChRs uses ligand-gated channel [30, 33, 34] and contributes to cognitive process [33, 35]. After a normal transmission of the neural signalling, the ACh binds with AChE [30, 36, 37] and gets degraded to acetate and choline (Figure 2). Each AChE molecule degrades about 25,000 molecules of ACh per second [38]. The ACh sits on lipophilic, uncharged anionic site of AChE with an electrostatic interaction [39, 40]. In this process, the amino acid Trp84 plays a vital role [41]. However, this set-up is inhibited reversibly and irreversibly by OPCs. The OPCs bind with AChE to form a complex (OPC, enzyme complex) which hinders the ACh hydrolysis, causing excessive cholinergic effect and rapid death within minutes. The intermediate syndrome has been reported to occur before 24 hours after exposure as well as, after 96 hours after exposure of OPCs [42]. This syndrome has been speculated to be related to the myopathy accompanied by weakness in respiratory muscles [43]. During respiratory complications, alveolar fluid, bronchorrhea acute respiratory disorder, aspiration pneumonitis, and pneumonia have also been reported [44].

Sometimes removal of a functional group from the OPCs such as nerve agents results in a stronger and permanent bond between the nerve agent and enzyme resulting in an irreversible inactivated state of enzyme, and this scenario is called ageing of the enzyme. The pace of enzyme ageing greatly varies with different nerve agents [45]. The serine residue of the catalytic site of cholinesterase [46] plays a vital role in ageing. Theoretically, it has been seen that Novichok also binds with acetylcholinesterase (AChE, E.C. 3.1.1.7) and leads to ageing of the enzyme [20, 47]. The stereochemistry of nerve agents also determines the extent of reactivation of the AChE [11]. Organophosphorus pesticides are also potent inhibitors of neuropathy target esterase (NTE). It has been reported in rats that dichlorvos decreases the Km value of NTE and, therefore, reduces the affinity of the enzyme for its substrate phenyl valerate [48].

### 3.2. Noncholinergic Effects

Some OPCs also cause another neurotoxicity known as organophosphate-induced delayed polyneuropathy (OPIDP) [49]. These toxicities result in the tingling effect of hands and feet, subsequently sensory loss, muscular weakness, and flaccidity of distal skeletal muscles, and ataxia [50, 51], after 2-3 weeks of exposures. OPIDP is also categorized as distal sensorimotor axonopathy [52] due to the inhibition of NTE [53]. NTE consists of both phospholipase and esterase activities [54], and belongs to patatin-like phospholipase domain-containing proteins (PNPLAs1-9) [55]. Mutations in NTE (PNPLA6) has been linked with the CNS and PNS alteration leading to congenital disorders [56]. OPIDP to occur about 70% of NTE inhibition has been seen to be necessary in the chicken [57]. Apart from inhibiting esterase, OPCs such as dichlorvos also showed inhibition of acyl peptide hydrolase which is a peptidase in a rat model [58]. The Inhibition of encephalin metabolism [59], endocannabinoid metabolism system [60, 61], and many lipases by OPCs [62] have also been reported. In addition, these compounds are also associated with oxidative stress which has been reported in many *in vivo, in vitro*, and human studies [63, 64].

## 4. Treatment Strategies for Organophosphorus Poisoning

**Figure 3:**
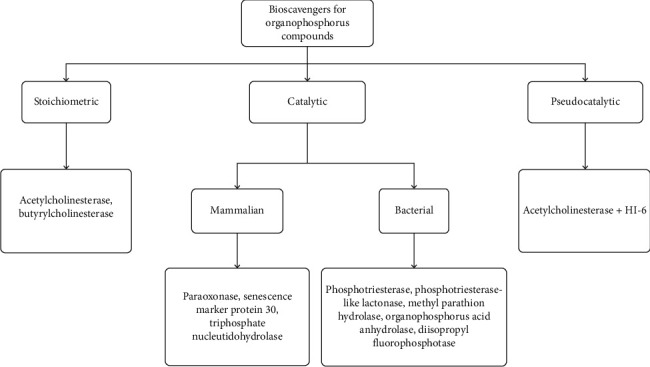
Classification of bioscavengers for organophosphorus compounds. The flowchart represents the classification of OPC hydrolysing enzymes on the basis of their hydrolysing ability into three categories, namely, stoichiometric, catalytic, and pseudocatalytic with their examples. The catalytic bioscavengers are further divided into two groups, bacterial and mammalian.

Although different antidotes are employed to treat the nerve agent poisoning, yet proficient recovery of aged AChE has been a challenge. Intravenously or intramuscularly introduced bioscavengers interact with OPCs and lead to neutralization reaction within bloodstream [65]. These can also be incorporated into the skin as active topical skin protectant [66] or have also shown neutralization effect to prevent OPC poisoning in macaques when introduced as aerosols or intravenously [67, 68]. Generally, bioscavengers can be divided into three types: (1) stoichiometric enzymes which bind with the OPCs and result in inactivation; (2) catalytic bioscavenger which hydrolyses the OPCs and degrades them to nontoxic components; and (3) pseudocatalytic bioscavengers which are a mixture of stoichiometric bioscavengers with a chemical reactivator such as oxime [69] (Figure 3).

**Figure 4:**
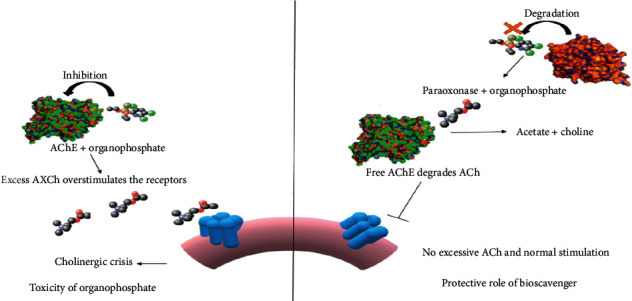
Diagrammatic representation of toxicity by OPCs and their detoxification. During the toxicity by OPCs, the compounds inhibit AChE and lead to excessive cholinergic effect, whereas the bioscavengers which can act as prophylactic agents neutralize the OPCs before they reach their targets and result in normal physiological hydrolysis of ACh and thus control proper signalling process.

There are many reactivators which are used to reactivate an AChE-OPC complex such as pralidoxime (PAM-2), trimedoxime (TMB-4), obidoxime (LuH-6, toxogonin) [10], and HI-6 (asomine, 1-(2′-hydroxyiminomethy-1′ pyridinum)- 3-(4′-carbomonyl-1-pyridinium)) [11]. For an enzyme to become a potential bioscavenger, it must overcome a number of impediments. It should have a high bimolecular rate and a large spectrum of activity with more enantiomeric preferences toward the toxic isomers. It should have long mean residence time in the body. It should not have an iatrogenic effect or/and any contaminants in it. It should also be easily available from natural sources, thermostable and economically affordable [70]. Naturally occurring enzymes which have organophosphatase activity as one of their promiscuous nature have been used as bioscavengers. These enzymes provide a prophylactic effect and neutralize the OPCs before they reach their target (Figure 4) in the blood [26]. Hence, different types of catalytic bioscavengers have been discussed below in detail.

### 4.1. Catalytic Bioscavengers

The stoichiometric bioscavengers are single-use enzymes with no turnover as one molecule of it binds with a single OPC for neutralization. Hence, the cost/dose of these enzymes is high compared to which the catalytic bioscavengers are multiple-time enzymes showing turnover and thus have low dose and high efficiency [26, 70]. However, unlike stoichiometric bioscavengers, some catalytic bioscavengers exhibit stereospecificity which limits the complete degradation of OPCs. Catalytic bioscavenger can be categorized as (1) bacterial type which includes enzymes such as methylparathion hydrolase, organophosphate hydrolase, and phosphotriesterase, and (2) mammalian type which includes enzymes such as human senescence marker protein 30, paraoxonase, and human triphosphate nucleotidohydrolase. Furthermore, the catalytic efficiencies, specificity, and mechanism of some of these catalytic bioscavengers are being discussed here.

#### 4.1.1. Bacterial Catalytic Bioscavenger

*(1) Phosphotriesterase (PTE)*. Phosphotriesterase (PTE) was first isolated from soil bacterium *Flavobacterium* sp. and *Pseudomonas diminuta* [71]. This protein belongs to the amidohydrolase superfamily and is a homodimeric metalloprotein with a TIM barrel structure (*α*/*β*) 8 folds [72], and each of its monomers consists of two zinc metal ions. Among the zinc metal ions, the one buried deeper in the structure is the *α* metal and coordinates with Asp301, His55, and His57, whereas the other known as the *β* metal is exposed to solvent and coordinates with His201 and His230. The zinc ions are bridged by a water molecule and Lys169 residue [73]. The residues associated with a small pocket are Gly60, Leu303, Ser308, and Ile106, while the large pocket is defined by His257, His254, Met317, and Leu271. Phe306, Phe132, Trp131, and Tyr309 are the residues in the hydrophobic leaving pocket [72]. A mutant (His257Tyr/Leu303Thr) of bacterial PTE showed catalytic efficiency approximately 10 times more than wild type for sarin and cyclosarin, and of about 100 times more for soman. It also showed stereospecificity and enhanced activity toward the P(−) enantiomer of cyclosarin [74, 75].

*(2) Phosphotriesterase-Like Lactonase (PLL)*. PLL, the closest structural homologues to *Pd*PTE, belongs to the amidohydrolase protein family. It catalyses lactones such as acyl-homoserine lactones (AHLs) by hydrolytic cleavage of their intracellular ester bond, and shows phosphotriesterase activity for OPCs. This enzyme also has (*β*/*α*) 8-barrel fold structure with a divalent metallic centre, which governs the catalytic activity [76]. PLL has been isolated from aerobic bacteria such as *Pseudomonas pseudoalcaligenes* [77] and extremophiles such as hyperthermophilic bacteria [78, 79], and from archaea [80]. PLL from *Deinococcus radiodurans (dr*PLL) is thermostable, robust, and soluble in nature. Random mutagenesis, rational protein designing, and crucial screening have been applied to create variants of PLL with enhanced organophosphatase activity. Among all the mutants, *dr*PLL 10 (Tyr28Leu/Asp71Asn/Tyr97Phe/Glu101Gly/Glu179Asp/Val235Leu/Pro274Leu) showed about 2800-fold decrease in lactonase activity with *δ*-lactone and an increase in OPCs hydrolysis up to 6.9 × 10^4^ fold [76]. Likewise, another thermostable lactonase from *Geobacillus kaustophilus* HTA426 (GkaP) after site-specific mutagenesis showed increased OPC catalysis. The mutant GkaP (Phe28Ile/Tyr99Leu/Thr171Ser/Phe228Leu/Asn269Ser/Val270Gly/Trp271Cys/Gal273Asp) showed an increase in catalytic efficiency by approximately 230 folds [79]. Another PLL *Sso*Pox isolated from *Sulfolobus solfataricus* has shown a promising OPC degrading capacity [25].

*(3) Methyl Parathion Hydrolase (MPH)*. Bacterial methyl parathion hydrolase (MPH, EC 3.1.8.1) was first identified in *Plesiomonas* sp. strain M6, which uses methyl parathion as its sole C and N sources [25, 81]. It consists of a dimeric structure with each subunit having mixed hybrid binuclear zinc. The ions are coordinated with His147, His149, His152, Asp151, His302, and His234 residues, and a water molecule. Asp255 and water molecule make a bridge between the zinc ions. The catalytic centre contains Phe196, Trp179, and Phe119 at the entrance [82]. Various recombinant MPHs have shown to be highly active on hydrolysing methyl parathion and other OPCs such as dichlorvos or malathion [83]. OPHC2 similar to MPH has been isolated and identified from *Pseudomonas pseudoalcaligenes strain* C2-1 [84] and *Stenotrophomonas strain* SMSP-1 [85]. Random mutation with MPH from *P. stutzeri* resulted in a 5-fold increase in chlorpyrifos hydrolysis, whereas the variant *Po*OPHm2 showed 6962- and 106-fold increases for methylparathion and ethyl paraoxon [86, 87], respectively.

*(4) Organophosphate Acid Anhydrolase (OPAA)*. OPAA (EC. 3.1.8.2) from *Alteromonas* sp. [73, 88] with phosphotriesterase and prolidase activity is a tetramer that is a dimer of a dimer structure [89, 90] and the functional oligomeric state of the enzyme. Each of the dimer subunits consists of a C-terminal with pita bread fold and N-terminal globular domain. The C-terminal consists of binuclear Mn^2+^ at the cleft. The solvent-exposed metal is ligated with His336 and Glu381, whereas the other metal is ligated in divalent orientation to Asp244, and these two metals are bridged by two other carboxylate ligands, Glu420 and Asp255. OPAA has prolidase activity specific to dipeptides with the second residue as Proline [73]. Its binding site comprises of three pockets: small, large, and leaving. The small pocket is lined with Try212, Val342, and His343, and capped with Asp45′ at N-terminal domain of the opposite subunit of dimer. The large pocket is associated with Leu225, His226, His332, and Arg418, and capped with Trp89′ from another subunit, and the leaving pocket consists of Tyr292 and Leu366 [73, 74, 91]. The proposed mechanism for OPAA enzymes states the presence of hydroxide bridge between two manganese (II) ions which start the nucleophilic attack on the phosphorus centre leading to the formation of an intermediate compound which then departs with the leaving group. His343 forms H-bonds with free phosphoryl oxygen and assists the positioning of the substrate [25, 73, 74].

*(5) Diisopropyl Fluorophosphatase (DFPase)*. Diisopropyl fluorophosphatase (DFPase, EC 3.1.8.2) from *Loligo vulgaris* squid is a phosphotriesterase that hydrolyses various OPCs [92]. It is a 35 kDa protein with a six-bladed *β* propeller structure [93, 94]. It has two calcium ions: one is responsible for structural integrity, and the other is responsible for the catalytic reactions. Asp229 is crucial for the activity of this enzyme, whereas the Glu21 is needed for calcium binding [95]. It hydrolyses P-F bond in the OPCs such as DFP molecule and releases the fluoride and diisopropylphosphate which is nontoxic. In this process, the fluorophosphate compound gets polarized by calcium ion stimulating attack by the carboxylate group and results in the fluoride release [93, 94, 96, 97].

#### 4.1.2. Mammalian Catalytic Bioscavenger

*(1) Paraoxonase (PON)*. The paraoxonase (E.C. 3.1.8.1.) family of proteins has three genes, PON1, PON2, and PON3, located on chromosome 7 in human (7q21.3-22.1), and chromosome 6 in the mouse. There is a 60% similarity in amino acid, and 70% in the nucleotide sequence within this family. PON1 with 355 amino acid weighs 43–47 kDa and has a cylindrical *β*-propeller feature with *β* strands linked by a disulphide bridge [98–100]. Two calcium ions are present at the centre, of the protein, one of which helps in catalytic activity, and the other maintains the structural stability of the enzyme [101, 102]. An apoprotein A-I stabilizes the association with HDL and enhances lactonase activity [98, 103]. When associated with HDL, it helps in lowering the oxidation of LDL [98, 103, 104]. It is mainly synthesised in the liver and secreted to plasma, but it is also present in the brain, small intestine, and kidney [98, 99, 105]. PON2 is associated with an inflammatory reaction, antioxidant property, and plays a role in defending against atherosclerosis. It is found intracellularly in tissues such as the lungs, placenta, testes, and heart, and mainly in the liver [98, 106, 107], whereas PON3 is found in the kidney and liver and displays antioxidant and antiatherosclerotic effect [98, 108]. PON proteins are related to various hydrolytic activities, namely, lactonase activity, arylesterase activity, and organophosphatase activity. Among all the PON proteins, PON1 exhibits all the three activities, whereas PON2 is linked with only lactonase activity, and PON3 shows lactonase and arylesterase activities [98, 104, 109].

PON1 gene is highly polymorphic with 184 SNPs, which includes 8 in the promoter region and 174 in the gene sequence [110, 111]. PON1 192 Gln/Arg and 55 Met/Leu polymorphisms have been associated with many health risk factors. It has been reported that homozygous 55 Leu/Leu shows lower LDL than heterozygous 55 Leu/Met, while 192Arg/Arg shows lower HDL and higher LDL compared to 192Gln/Gln [98]. Homozygous 192Arg/Arg and 55Met/Met are associated with high levels of thiobarbituric acid-reactive substances, conjugated dienes, and low level of GSH in blood, whereas the homozygous counterparts, i.e., 192Gln/Gln and 55Leu/Leu, showed completely opposite effect, testifying that 192Gln/Gln and 55Leu/Leu have a protective role against oxidative stress [112]. Some other polymorphisms such as PON2 148Ala/Gly and PON3 311Cys/Ser, and polymorphisms in the promoter region such as 107Cys/Thr, 162Ala/Gly, and 907Gly/Cys [110] of PON1 have been studied for their relationship with heart diseases. Homozygous PON1 107Cys/Cys has been reported to be associated with high HDL [98, 113].

*(2) Senescence Marker Protein 30 (SMP30)*. Human senescence marker protein 30 (HuSMP30; EC 3.1.1.17) is a 34 kDa protein [114] with a six bladed *β*-propeller structure which consists of a single metal ion binding centre. It is primarily expressed in the liver and kidney tissues. It is found in the lungs, kidney, liver, testes, and cerebrum, and is conserved in the vertebrates [115]. In humans, it is located at p11.3-q11.2 of the X chromosome [114]. Like PON1, it has also been reported to show lactonase [116] activity as its physiological function; however, it also catalyses the hydrolysis of an OPC, diisopropyl fluorophosphate, thus acting as DFPase [117]. SMP30 is also referred to as regucalcin due to its high affinity with calcium indicating its role in maintaining calcium homeostasis in the cells [118–120]. The penultimate step of ascorbic acid biosynthesis in nonprimates is catalysed by SMP30 [121]; however, the ascorbic acid biosynthesis pathway is not functional in human. These analyses suggest that SMP30 might play an important role in calcium homeostasis and providing protection against OPC poisoning in human. A recent study from our research group demonstrated that both mouse and human SMP30 showed organophosphate hydrolase activity in the presence of Ca^2+^ and Zn^2+^, thus displaying metal-dependent promiscuous activity [116]. A recombinant HuSMP30 has also been expressed with chaperones as a soluble protein and has been characterized for its DFPase activity [122].

*(3) Human Triphosphate Nucleotidohydrolase (dUTPase)*. Human triphosphate nucleotidohydrolase (dUTPase; EC: 3.6.1.23) is a trimerically structured enzyme with a 3-fold axis. The subunits of this protein interact through twisted *β*-sheets thus resulting in burying the hydrophobic surfaces about the axis [123]. It converts deoxyuridine triphosphate to deoxyuridine monophosphate [124] and prevents DNA uracilation, and maintains genome integrity [125]. It consists of three active sites and requires divalent Mg^2+^ for its activity. It is a potent enzyme and has shown to have a stereoselective preference toward P(−) enantiomer of VX [126].  Table 1 contains the list of variants of these organophosphatase and their compared activity with their respective wild types.

### 4.2. Stereoselective Degradation

Enzymes have the ability to differentiate between the enantiomers of the substrate, and this ability is termed as the stereospecificity of an enzyme. This is a vital property of a biocatalyst; it allows the biocatalyst to undergo either fast or slow reaction on the basis of the reaction orientation. This property is exploited in chemical, pharmaceutical, and agrochemical industries [141]. Since the naturally occurring enzymes have inefficient stereoselectivity/regioselectivity and thermostability, they have not been able to achieve eminence in practical procedures. The emergence of protein engineering techniques such as direct evolution has proven to be a vital tool in engendering various genetic amendments to construct variants of natural enzymes with superior activity compared to their wild types [128, 140–143].

**Figure 5:**
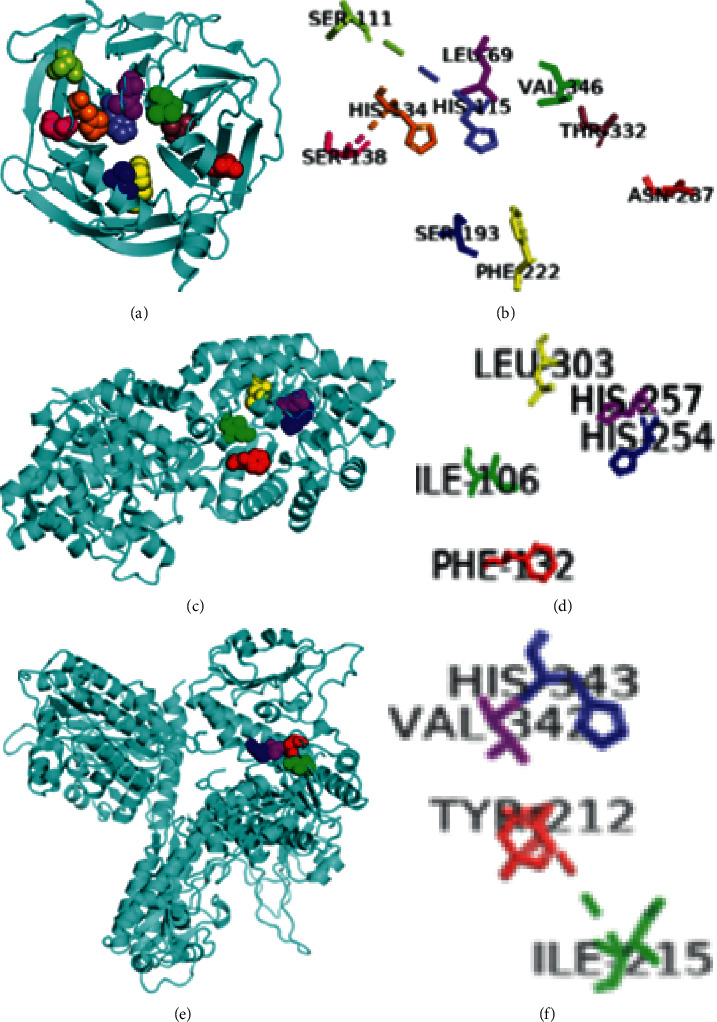
Illustration represents residues important for stereoselectivity of the enzymes: (a) PON1 cartoon structure with different coloured sphere-shaped residues; (b) PON1 residues as sticks; (c) PTE cartoon structure with different coloured sphere-shaped residues; (d) PTE residues as sticks; (e) OPAA cartoon structure with different coloured sphere-shaped residues; (f) OPAA residues as sticks. (For creating the above structures, basic structures were taken from PDB ID: 1V04, 3CAK, and 3L7G, and PyMOL software was used.)

A special substrate asymmetrical fluorogenic organophosphate, CMP-MeCyC, was created and used to study the stereoselective hydrolysis by mammalian wild-type PON1 and its variants, in which a variant 4.27/V346A (Leu69Val/Ser138Leu/Ser193Pro/Asn287Asp/Val346Ala) showed the highest detoxification. The wild type of PON1 displayed a preference for the P (+) isomer, whereas the mutated variant of PON1 showed more active hydrolysis toward P(−) isomers of CMP-MeCyC [144]. In another experiment, mutant with H115W mutation exhibited 270–380-fold increase in hydrolytic activity for parathiol, whereas mutant with Leu69Val results in 10–100-fold increase in activity for DFP, cyclosarin, and soman. PON1 variants with Val346Ala, Leu69Val/Ser193Pro/Val346Ala, and Leu69Val/Ser138Leu/Ser193Pro/Asn287Asp/Val346Ala have resulted in 4- to 10-fold increase in the hydrolysis of ChPo and P-F containing organophosphorus esters [145]. Another variant 4E9 (Leu69Gal, Ser111Thr, His115Trp, His134Arg, Phe222Ser, Thr332Ser) has shown to be an active variant in hydrolysing P(−)-CMP-coumarin and P(−)-CMP-F [130] (Figures 5(a) and 5(b)).

The large, small, and leaving pockets of enzyme PTE play a crucial role in the stereoselectivity of the enzyme and are manipulated by the mutation process to enhance, reverse, and relax this stereoselectivity. Decreasing the large pocket and enlarging the small pocket by the rational engineering of PTE invert the enantiomer selectivity of the wild type. The wild type prefers the hydrolysis of the less-toxic P (+) enantiomer of GB, GD, and GF, and their analogous, whereas the mutants GWT, GGY, and YT displayed preference toward P(−) enantiomer hydrolysis. The molecular dynamics simulation also revealed that the leaving group orientation plays an important factor for chiral selectivity. Mutant with mutation His254Gal/His257Trp/Leu303Thr showed a preference toward the P(−) enantiomers of sarin [72, 138]. Another mutant Ile106Ala/Phe132Ala/His257Tyr has also shown a high preference toward the P(−) enantiomer of sarin [72]. Mutant C74 with mutations His254Asn and Ile106Ala showed a reduction in P(+) isomer preference and increase in P(−) isomer preference for RVX [146] (Figures 5(c) and 5(d)). Similarly, OPAA mutant FLYD (Try212Phe/Val342Leu/Ile215Try/His343Asp) with four mutations showed 16-fold increase in catalysis compared to wild type, and broader stereospecificity for VR [74] (Figures 5(e) and 5(f)).

## 5. Conclusions and Future Perspectives

The indirect or direct exposures of various OPCs, especially the nerve agents, have proven to be an utmost important threat causing severe health issues. The quantity and the route of OPC exposure may lead to acute, intermediate, or delayed symptoms in humans. Although these chemicals are generally used extensively as pesticides and antiacetylcholinesterase for treatment purpose in diseases such as Alzheimer's, their long-term side effect proves to be a major concern. Majority of these chemicals are chiral compounds and are generally present in their different enantiomeric forms. Existing antidotes have limited efficiency due to which research for more effective treatment strategies is being carried out globally. This has led to manipulate the promiscuous nature of catalytic bioscavengers such as paraoxonase and PTE via protein engineering techniques to generate mutants with enhanced ability to degrade the OPCs before they reach their target niches. Techniques such as directed molecular evolution where different types of mutations are employed to generate mutants which have higher efficiency toward more toxic P(−) isomers, are thermostable and are able to hydrolyse a racemic mixture of OPCs effectively. These superior mutants with better delivery systems are potential OPC-hydrolysing agents in the near future.

## Figures and Tables

**Table 1:** List of mutants of organophosphatase and their activity in comparison to the wild-type enzymes.

Enzymes	Mutants	Kcat	Km	Kcat/Km	Activity compared to WT	References
PON1 3SRE*∗*, 1V04*∗*	His115Trp 4HHO^*∗*^	7.3 ± 0.2 s^−1^	1.6 ± 0.1 mM	4700 ± 246 M^−1^s^−1^	Increase in organophosphatase activity (paraoxonase activity)	[127]

PON1	3B3 (Asn41Asp, Ser110Pro, Leu2240Ser, His243Arg, Phe264Leu, Asn324Asp, Thr332Ala)	Not detectable (P(−)-CMP coumarin)	Not detectable (P(−)-CMP-coumarin)	<0.0002(1) *μ*M^−1^ (P(−)-CMP-coumarin)	Approx. 250-fold increase in catalytic efficiency	[128–130]

PON1	3D8 (Leu69Gly, His115Trp, His134Arg, Met196Val, Phe222Ser, Thr332Ser)	546 ± 29 min^−1^ (P(−)-CMP-coumarin)	36.7 ± 7 *μ*M (P(−)-CMP-coumarin)	12.7 ± 2 (>63,000) *μ*^−1^min^−1^ (P (−)-CMP-coumarin)	A similar rate of hydrolysis for P (+) and P(−) isomers	[130]

PTE	GWT-f5 (His254Gly/His257Trp/Leu303Thr/Met317Leu/Ile106Cys/Phe132Ile/Leu271Ile/Lys185Arg/Ile274Asn/Ala80Val/Arg67His) 3URB^*∗*^	1.2 × 10^2^ s^−1^ (analogues of cyclosarin P(−))	—	3.2 × 10^5^ M^−1^s^−1^ (analogues of cyclosarin P(−))	Best mutant for hydrolysis of cyclosarin analogues	[75]

PTE	L7eP-3a-I106G (His245Gln/His257Phe/Ile106Cys/Phe132Val/Ser308Leu/His257Tyr/Ala270Val/Leu272Met/Ile274Asn/Ile106Gly) 4ZSU^*∗*^	58 s^−1^ (P(+)-APVR), 166 s^−1^ (P(−)-APVR), 545 s^−1^ (paraoxon) not detectable (DEVX)	0.71 mM (P (+)-APVR), 0.31 mM (P(−)-APVR), 33 *μ*M (paraoxon) not detectable (DEVX)	8.2 × 10⁴ M^−1^s^−1^ (P (+)-APVR), 5.3 × 10^5^ M^−1^s^−1^ (P(−)-APVR), 1.67 × 10^7^ M^−1^s^−1^ (paraoxon), 7.7 × 10^3^ M^−1^s^−1^ (DEVX)	620-fold increase in catalytic activity for P (−)-VR type agents	[131]

PTE	His254Gly/His257Trp/Leu303Thr	—	—	—	Increased in turnover no. by 3 orders for a toxic isomer of soman	[132]

DFPase	Ser271Ala	—	—	—	30% increase in enzymatic activity for DFP	[133]

DFPase 1PJX*∗*	His287Phe,	14 s^−1^	0.03 ± 0.005 mM	—	Increase in activity for DFP	[134]

PTE-like lactonase 3FDK*∗*	Try28Leu/Asp71Asn/Tyr97Phe/Glu101Gly/Glu179Asp/V235Leu/Pro274Leu 4J35^*∗*^	6.8 × 10^4^ s^−1^	—	3.6 × 10^5^ M^−1^s^−1^	Enhanced activity with methyl phosphonates	[76]

OPH 1DPM*∗*	His254Arg, His257Leu	640 s^−1^ (paraoxon), 50 s^−1^ (demeton-S)	0.07 ± 0.01 mM (paraoxon), 6.2 ± 1.4 mM (demeton-S)	9.14 × 10^6^ M^−1^s^−1^ (paraoxon), 8.06 × 10³ M^−1^s^−1^ (demeton-S)	Increased catalytic activity for VX and VR	[135]

OPH 1DPM*∗*	His254Arg 1QW7^*∗*^	750 s^−1^ (paraoxon)	0.012 mM (paraoxon)	6.25 × 10^7^ M^−1^s^−1^ (paraoxon)	11.3-fold increase in activity	[136]

OPH	22A11 (Ala14Thr, Ala80Val,Lys185Arg, H257Y, Ile274Asn)	39600 ± 2100 s·s^−1^ (methyl parathion)	0.62 ± 0.02 mM (methyl parathion)	6.4 × 10^7^ M^−1^s^−1^ (methyl parathion)	25-fold increase activity for methyl parathion	[137]

OPH	Leu27Ile/Try309Ala	0.15 s^−1^	5.87 mM	24.9 M·s^−1^	150-fold increase activity for VX^a^	[138]

Prolidase	Tyr301Cys/Lys342Asn	2861 s^−1^	2.92 ± 0.40 mM	980 mM^−1^s^−1^	Thermostable and increase activity for soman	[139]

OPAA	FL (Try212Phe/Val342Leu)	6.9 × 10^4^ ± 3 × 10^3^ min^−1^ (GP), 6.6 × 10^4^ ± 3.4 × 10^3^ min^−1^ (soman), 4.52 × 10^4^ ± 4.62 × 10^3^ min^−1^ (sarin)	534.924 ± 67.8 *μ*M (GP), 883.16 ± 156.4 *μ*M (soman), 5.31 × 10^3^ ± 1 × 10^3^ *μ*M (sarin)	1.29 × 10^8^ ± 2.21 × 10^7^ M^−1^min^−1^ (GP), 7.48 × 10^7^ ± 1.71 × 10^7^ M^−1^min^−1^ (soman), 8.50 × 10^6^ ± 2.49 × 10^6^ M^−1^min^−1^ (sarin)	Increase in catalysis compared to wild type and preference to R (+) enantiomer of sarin	[140]

## Data Availability

All the relevant data used to support the findings of this study are included in the article.

## Abbreviations

OPCW: Organisation for the Prohibition of Chemical Weapons
OPCs: Organophosphorus compounds
ACh: Acetylcholine
AChE: Acetylcholinesterase
mAChRs: Muscarinic acetylcholine receptors
nAChRs: Nicotinic acetylcholine receptors
PON: Paraoxonase
PTE: Phosphotriesterase
OPAA: Organophosphate acid anhydrolase
OPIDN: Organophosphate-induced delayed neuropathy
NTE: Neuropathy target esterase
MPH: Methylparathion hydrolase
PLL: Phosphotriesterase-like lactonase
SMP30: Senescence marker protein 30
AHL: Acyl-homoserine lactone
ChAT: Choline acetyl transferase
PNPLAs: Patatin-like phospholipase domain-containing proteins
dUTPase: Human triphosphate nucleotidohydrolase.
